# Supplementary material for: Effect of integrated reporting quality disclosure on cost of equity capital in developed markets: Exploring the moderating role of corporate governance quality
Source: PLoS One. 2025 Jun 3;20(6):e0324147. doi: 10.1371/journal.pone.0324147 (PMC12133193; doi:10.1371/journal.pone.0324147)
Supplement: Attachment — Submitted filename: Response to Reviewers.docx [file pone.0324147.s002.docx]

**Manuscript number:** PONE-D-24-26051

**Title:** Effect of integrated reporting quality disclosure on cost of equity capital in developed markets: Exploring the moderating role of corporate governance quality

**Journal:** PLOS ONE

Dear Academic Editor,

Thank you for the comments concerning our manuscript. We deeply appreciate your positive evaluation of our work. Those comments are valuable and very helpful. We have read through comments carefully and have made corrections. Please see below; all tasks and revisions taken are shown point-by-point.

**Response to Academic Editor comments**

**Comments #1:** Please ensure that your manuscript meets PLOS ONE's style requirements, including those for file naming.

Response to comment #1: Thank you for your comment. We have carefully reviewed and ensured that the manuscript now fully complies with PLOS ONE's style requirements, including the file naming conventions. Please let us know if there are any additional adjustments required.

**Comments #2:** In this instance it seems there may be acceptable restrictions in place that prevent the public sharing of your minimal data. However, in line with our goal of ensuring long-term data availability to all interested researchers, PLOS’ Data Policy states that authors cannot be the sole named individuals responsible for ensuring data access (http://journals.plos.org/plosone/s/data-availability#loc-acceptable-data-sharing-methods).

Data requests to a non-author institutional point of contact, such as a data access or ethics committee, helps guarantee long term stability and availability of data. Providing interested researchers with a durable point of contact ensures data will be accessible even if an author changes email addresses, institutions, or becomes unavailable to answer requests.

Before we proceed with your manuscript, please also provide non-author contact information (phone/email/hyperlink) for a data access committee, ethics committee, or other institutional body to which data requests may be sent. If no institutional body is available to respond to requests for your minimal data, please consider if there any institutional representatives who did not collaborate in the study, and are not listed as authors on the manuscript, who would be able to hold the data and respond to external requests for data access? If so, please provide their contact information (i.e., email address). Please also provide details on how you will ensure persistent or long-term data storage and availability.

Response to comment #2: Thank you for your comment. We have uploaded a minimum data as a supplementary file.

**Comments #3:** The topic of this article is very important, but it is also a classic topic, so there is a wealth of relevant research literature. The author needs to better extract the innovative points of this article in the introduction and expand the inspiration of this article for existing research in the conclusion.

Response to comment #3: Thank you for your thoughtful and constructive comment. We have revised the introduction and conclusion sections to clearly articulate the innovative contributions of this study and to expand on its implications for existing research. Additionally, we have updated the literature review sections (2.1 and 2.2) to provide a detailed discussion of the research gap this study addresses. These revisions aim to better highlight the unique aspects of our work and its relevance to the existing body of literature. Please refer to the revised Sections 1, 2.1, 2.2, and 5 for these updates.

**Comments #4:** When estimating the IRQ on cost of equity capital in this article, the identification strategy needs to be more refined. Currently, it is difficult to avoid potential endogeneity issues, and some robustness results need to be supplemented.

Response to comment #4: Thank you for your insightful comment. To address the potential endogeneity issues, we have incorporated two econometric models—IV2SLS and system-GMM—under the "Robustness of the Results" section. These models were employed to ensure the reliability of our findings and mitigate endogeneity concerns. Please refer to the revised text in Sections 3.2.5 and 4.4 for further details.

**Comments #5:** This article refers to many references, but the summary of relevant literature in the past two years is insufficient, making it difficult to reflect the current research progress and trends. It is recommended that the author make structural adjustments in terms of references.

Response to comment #5: Thank you for your valuable suggestion. We have included the latest references relevant to our research context. Please refer to the revised manuscript for further details.

**Response to Reviewer #1 comments**

**Comments #1:** This article analyzes a topical issue: the relationship between the quality of integrated reporting (IRQ) and the cost of capital, including the effect of corporate governance quality (CGQ). Authors rely on agency theory and argue that to reduce information asymmetry, firms issue more information which allows to reduce the cost of capital. Therefore, a more comprehensive firms’ reporting, including information about their strategy and risks, increases investors’ confidence, hence it may reduce the cost of capital. According authors their contribution consists of analyzing the role of information about employees. The structure of the article is correctly chosen and it exhibits a logical concern.

Response to comment #1: We sincerely thank the reviewer for the thoughtful and encouraging feedback on our manuscript. We appreciate your recognition of the topical nature of our study and its reliance on agency theory to examine the interplay between integrated reporting quality, corporate governance quality, and the cost of capital. Your acknowledgment of the logical structure and clear exposition of our argument is particularly gratifying. Additionally, we are pleased that you noted our emphasis on the role of employee-related information in our analysis. We believe this aspect adds a unique dimension to understanding how integrated reporting enhances transparency and reduces information asymmetry, ultimately contributing to reducing the cost of capital. Thank you once again for your constructive comments, which is undoubtedly strengthen the quality of our work.

**Comments #2:** In introduction authors mentioned stakeholders and shareholders on line 66. I suggest to remove shareholder or including “in particular” because shareholders are stakeholders too.

Response to comment #2: We thank the reviewer for the insightful comment regarding the phrasing on line 66. We have revised the sentence to clarify the relationship between stakeholders and shareholders, acknowledging that shareholders are a subset of stakeholders. The revised sentence now reads: "*Stakeholders, particularly shareholders, now regularly demand comprehensive financial information.*"

**Comments #3:** Regarding literature review, it is not clear what authors add to previous literature given that the relation between IRQ and Cost of Capital and the influence of CGQ has been studied before, for example De Villiers et al. (2017), Pavlopoulos et al. (2017) and Gupta et al. (2018).

Response to comment #3: Thank you for your valuable feedback. We have revised Sections 2.1 and 2.2 of the literature review to incorporate the research gap addressed in our manuscript. The updated sections now provide a clearer articulation of the gap and its significance to our study. Kindly refer to the revised Sections 2.1 and 2.2, as detailed in the text below:

*“2.1 Relationship between IRQ disclosure and ICC*

*Integrated reporting has gained attention as a mechanism for improving financial transparency and aligning corporate governance with stakeholder expectations (de Villiers, Venter and Hsiao, 2017; Pavlopoulos, Magnis and Iatridis, 2017). By integrating financial and non-financial disclosures, IR aims to enhance decision-making, reduce information asymmetry, and ultimately lower the cost of capital. Non-financial data, including CSR initiatives, have been observed to correlate with the cost of equity capital. Breuer et al. (2018) investigated the link between CSR, investor protection, and equity capital costs, noting that in jurisdictions with robust investor safeguards, CSR investment tends to lower equity costs, whereas in contexts with weaker investor protection, such investments tend to elevate equity costs.*

*Theoretical frameworks, as elucidated by Dhaliwal et al. (2011), underscore the significance of employing prospective information to gauge equity capital costs. Voluntary disclosure of non-financial data is viewed as a means to bridge the gap between external and internal information, thereby diminishing equity capital costs and attracting long-term investors. Moreover, following the mandatory adoption of international financial reporting standards (IFRS), there was an enhancement in accounting information quality (Barth, Landsman and Lang, 2008; Armstrong, Guay and Weber, 2010; Devalle, Onali and Magarini, 2010), leading to a subsequent reduction in capital costs (Lee and Yeo, 2010; LI, 2010; Hoque, 2017). Enhanced information quality is associated with fewer instances of managerial misappropriation, thereby resulting in lower equity capital costs (Zhou, 2014). Nonetheless, certain studies have identified a negative correlation between voluntary non-financial information disclosure and equity capital costs (Griffin and Sun, 2013; Plumlee et al., 2015).*

*Integrated reporting represents a voluntary reporting framework that holds promise for revolutionizing corporate reporting practices (Williams and Lodhia, 2021). Recent data from a survey conducted by PricewaterhouseCoopers (PwC) reveals that approximately two-thirds of investment professionals perceive the quality of a company's reporting, encompassing details about strategy, risks, and other value drivers, as directly influencing the cost of capital (Songini et al., 2023). Nonetheless, a dearth of studies exists investigating the nexus between IRQ and the capital market, warranting further exploration in this domain to enhance comprehension of integrated reporting's impacts on the cost of equity capital.*

*Prior studies have explored the relationship between the quality of financial disclosures and a firm’s cost of capital (Gupta, Krishnamurti and Tourani-Rad, 2018). Research using global datasets analyzed IR and capital costs across 27 countries, covering 995 firms and 3,294 observations from 2009 to 2013 (García-Sánchez and Noguera-Gámez, 2017; Zhou, Simnett and Green, 2017). Maria and Ligia’s (2017) found a negative association between IRQ disclosure and the cost of capital but noted that differences in corporate governance systems among countries did not significantly influence this relationship. In contrast, Barth et al. (2017), studying 80 firms listed on the Johannesburg Stock Exchange, found no significant relationship between IRQ and ICC. Similarly, Lee and Yeo (2016) observed a positive association between IRQ disclosure and firm valuation, contributing to mixed findings on the IRQ-ICC relationship.*

*Existing literature also highlights limitations. For instance, while de Villiers et al. (2017) primarily focused on the conceptual underpinnings of IR, emphasizing its potential to provide a comprehensive narrative of value creation but lacking empirical insights into its impact on cost of capital. Pavlopoulos et al. (2017) identified a positive association between IR quality and corporate governance but did not explore how this interaction affects financial outcomes like cost of equity. Similarly, Gupta et al. (2018) demonstrated the significance of governance mechanisms and financial development in reducing cost of equity but did not examine IR as a specific governance tool. This leaves a critical gap in understanding how IRQ interacts with CGQ to influence a firm’s financial outcomes, particularly within different institutional contexts.*

*Our study aims to address this gap by investigating the direct and interactive effects of IRQ and CGQ on the cost of capital. Unlike prior research, our approach explicitly incorporates the dynamic interplay between these variables, offering insights into their complementary roles across diverse regulatory and financial environments. By doing so, this study contributes to a more nuanced understanding of the mechanisms through which IR impacts financial metrics, expanding the scope of existing literature.*

*2.2 Relationship among IRQ disclosure, CGQ, and ICC*

*Previous investigations have provided empirical support for the advantages associated with the adoption of high-quality integrated reporting (IIRC, 2013; de Villiers, Rinaldi and Unerman, 2014; Adrián-Martínez et al., 2016; Hoque, 2017). However, an exclusive focus on IRQ may not furnish a comprehensive understanding of its influence on capital market outcomes, particularly its efficacy on the cost of capital, without accounting for a robust institutional framework. Accounting practices, as evidenced in prior studies, were shaped by diverse factors, including the underlying capital markets (Walker, 2010; Wysocki, 2011; Cieslewicz, 2014; Wehrfritz and Haller, 2014). While certain studies have suggested a tenuous relationship between IRQ disclosure and ICC (Lee and Yeo, 2016; Barth et al., 2017; García-Sánchez and Noguera-Gámez, 2017; Zhou, Simnett and Green, 2017), there exists a correlation between an integrated reporting framework and the capital market (Zhou, 2014).*

*Nonetheless, extant research has predominantly concentrated on the impact of high-quality disclosure on the capital market, overlooking other institutional variables, despite theoretical assertions regarding the synergy between high-quality reporting disclosure and other institutional factors. Previous study has illustrated that CGQ has been employed as a moderating variable in numerous investigations (Chen and Paulraj, 2004; Alhazaimeh, Palaniappan and Almsafir, 2014; Ould Daoud Ellili, 2020; Abu Alia et al., 2022; Tawfik et al., 2022; Liu, Li and Lin, 2023).*

*Our research endeavors to bridge this void by exploring the influence of corporate governance on both the IRQ disclosure and ICC. Through the examination of employees' role in CGQ as a moderating variable, we aim to offer valuable insights into how governance practices shape the relationship between IRQ disclosure and ICC. In this segment, we delve into the correlation between IRQ disclosure and ICC (Section 2.1). Subsequently, we broaden our investigation to encompass the association between IRQ disclosure, CGQ, and ICC (Section 2.2). Previous studies have already scrutinized the nexus between voluntary disclosure, encompassing both financial and non-financial aspects, and corporate governance (Hail and Leuz, 2006; Fifka and Pobizhan, 2014; Ortas et al., 2015; Haldar and Raithatha, 2017).*

*A pioneering approach to enhancing CSR reporting is IR, which endeavors to amalgamate CSR disclosures (Sun et al., 2022). Furthermore, scholars have noted a positive linkage between CGQ and CSR, with Huang (2010) documenting a robust correlation. Similarly, in emerging economies, Jahid et al. (2022) have affirmed the influence of CGQ practices on CSR disclosures. However, Sriani and Agustia (2020) identified no significant association between IRQ and information asymmetry as measured by the spread. The ESG information is pivotal in evaluating companies' risk profiles, future cash flows, and effectively pricing investments (Veltri et al., 2023). Institutional factors, such as corporate governance, directly shape voluntary disclosure and its ramifications on equity costs (Reverte, 2009; Pavlopoulos, Magnis and Iatridis, 2017; Gupta, Krishnamurti and Tourani-Rad, 2018; Mohamed Adnan, Hay and van Staden, 2018).*

*Hence, drawing upon extant literature, we envisage that CGQ assumes a pivotal role in fortifying the relationship between IRQ disclosure and ICC in developed markets. De Villiers et al. (2017) introduced IRQ as an approach to reporting that encompasses both financial and non-financial information within a unified report. Subsequent extensive research has investigated the correlation between IRQ disclosures, CGQ, and ICC. Consistently, studies have revealed a positive association between IRQ disclosure and these variables (Haldar and Raithatha, 2017). Pavlopoulos et al. (2017) contend that high-quality disclosure via integrated reporting fosters more effective corporate governance mechanisms. Consequently, this influences companies' investment decisions, impacting the anticipated cash flow ratio and ultimately influencing the cost of equity capital (Skaife, Collins and LaFond, 2006).*

*Indeed, effective CGQ plays a pivotal role in mitigating both agency costs and ICC (Reverte, 2009). Corporate governance mechanisms are crafted to address agency dilemmas and ensure managerial actions align with shareholders' best interests (Hidalgo, García-Meca and Martínez, 2011). The theoretical underpinning of the nexus between corporate governance and disclosure stems from agency theory, which tackles issues of information asymmetry and accountability, thereby resulting in a reduction in the cost of equity capital. Through the implementation of robust corporate governance practices, firms can bolster transparency and accountability, consequently diminishing information asymmetry between managers and shareholders. This engenders increased confidence among investors and analysts, positively shaping perceptions regarding the firm's value and future prospects. Consequently, the ICC diminishes as investors perceive the firm as less risky, thereby demanding a reduced return on their investment.*

*When integrated with robust CGQ, IRQ disclosure can reinforce these favorable outcomes. IRQ disclosure, encompassing both financial and non-financial information, offers a comprehensive overview of a firm's performance and sustainability endeavors. This can lead to enhanced decision-making, heightened stakeholder confidence, and improved long-term strategizing, all contributing to the reduction of the ICC. Overall, the research underscores the notion that a combination of robust CGQ and IRQ disclosure can exert a positive influence on the ICC. Through augmenting information quality, aligning managerial and shareholder interests, and fostering transparency, firms can cultivate a conducive investment environment that attracts investors and ultimately diminishes the ICC.*

*The literature consistently highlights the interconnection between CGQ and ICC, with various conceptualizations of corporate governance demonstrating an influence on the ICC. Empirical investigations consistently indicate that firms implementing stronger governance practices experience diminished capital costs (Ashbaugh-Skaife, Collins and LaFond, 2006). Furthermore, Gupta et al. (2018) evidenced that firm-level CGQ amplifies the effect on the ICC. Conversely, Boubakri et al. (2016) observed that distant privatized firms in countries characterized by weak institutional governance encounter elevated ICC. Zhou (2014) unveils that firms boasting robust CGQ incur lower costs of equity, particularly in nations with robust legal frameworks, sound government quality, and extensive disclosure standards. Jebran and Chen (2023) analyze how corporate governance practices aided firms in navigating the challenges posed by the COVID-19 pandemic. Specifically, Pavlopoulos et al. (2017) establish a positive correlation between IR and CGQ. Nonetheless, it is noteworthy that the literature presents mixed findings regarding the relationship between CGQ and ICC (Chen, Chen and Wei, 2009; Reverte, 2009; Gupta, Krishnamurti and Tourani-Rad, 2018).*

*Given this diversity in results, CGQ plays a pivotal role in influencing a firm’s cost of capital by enhancing transparency, mitigating agency conflicts, and improving investor confidence (Gupta, Krishnamurti and Tourani-Rad, 2018). Existing literature highlights that strong governance practices, such as board independence and robust shareholder rights, contribute to lower capital costs, particularly in regions with developed financial systems. Pavlopoulos et al. (2017) extended this discourse by demonstrating that firms with higher IRQ exhibit superior governance mechanisms, which further reduce agency costs. However, they did not explore how these governance improvements mediate the relationship between IRQ and cost of capital. Gupta et al. (2018) emphasized the complementary effects of country-level financial development and firm-level governance in reducing cost of equity, underscoring the importance of external institutional environments. Building on this, we argue that IRQ can act as a catalyst for enhancing CGQ, thereby amplifying its impact on financial outcomes. While prior studies have largely treated CGQ and IR as independent constructs, this study examines their interaction, hypothesizing that IRQ enhances CGQ’s effectiveness in reducing cost of capital by fostering integrated decision-making and improving the quality of disclosures. By investigating these interdependencies, this study not only fills a significant gap in the literature but also provides actionable insights for policymakers and practitioners seeking to leverage IR as a tool for improving governance and financial performance. The findings are expected to inform strategies for aligning IR and CGQ to achieve optimal financial outcomes, particularly in emerging markets where governance and disclosure practices are still evolving.”*

**Comments #4:** Authors use a unique variable to estimate CGQ which may imply loosing relevant information about different mechanism of corporate governance. It would be beneficial to support their argument using other studies showing that it brings transparency and captures stakeholders’ confidence. In addition, the definition of the control variable size should be presented in the text (market capitalization, ln total assets, or other) as well as the definition of Lev.

Response to comment #4: We acknowledge the reviewer’s concern regarding the potential loss of information by presenting CGQ as a singular variable. However, the decision to aggregate the four governance categories into a single metric is supported by prior literature (Ashbaugh-Skaife et al., 2006; Aggarwal et al., 2009; Reverte, 2009; Pavlopoulos et al., 2017; Gupta et al., 2018; Al-Gamrh et al., 2024; Honey et al., 2025). Studies such as Ashbaugh-Skaife et al. (2006) and Gupta et al. (2018) have demonstrated that unified governance metrics effectively capture the overall quality of corporate governance, providing a transparent and comprehensive measure that aligns with investor priorities. Moreover, previous research has highlighted that simplified governance measures enhance transparency and stakeholder confidence. For instance, Zhou (2014) argues that aggregated governance scores facilitate comparability across firms, which is essential for investors and regulatory bodies. Similarly, Aggarwal et al. (2009) emphasize that a unified governance metric provides an actionable tool for stakeholders to evaluate governance effectiveness, thereby reducing information asymmetry and fostering trust. While disaggregated measures may provide more granular insights, they can introduce complexities in interpretation and limit the ability to evaluate governance holistically (Hail & Leuz, 2006). By consolidating the four categories, our approach acknowledges the interdependence of governance dimensions and delivers a practical tool for assessing overall governance performance, consistent with prior studies (Reverte, 2009; Pavlopoulos et al., 2017). We have revised this section accordingly. Please find the updated text below:

*“3.2.2 Measurement of moderating variable*

*In this research, we employed a methodology akin to that utilized by Aggarwal et al. (2005; 2009) and Gupta et al. (2018) to assess CGQ as an explanatory factor. Specifically, we relied on institutional shareholder services (ISS) risk metrics to appraise a firm's performance, drawing from regulatory filings, annual reports, and website content. ISS has devised a comprehensive rating framework encompassing four overarching categories: anti-takeover measures, audit practices, compensation and ownership structures, and board effectiveness (Aggarwal et al., 2009; Alhazaimeh, Palaniappan and Almsafir, 2014). Subsequently, we devised individual ratings for each of these categories, comprising board composition and efficacy, anti-takeover provisions, director and executive remuneration as well as ownership structures, and audit methodologies. These ratings were amalgamated to derive an overarching score for each firm, consistent with the methodology adopted by Aggarwal et al. (2005; 2009).*

*Presenting CGQ as a singular variable, rather than delineating it across four categories, offers several advantages. Firstly, it streamlines the analysis and facilitates result interpretation by furnishing a unified score encapsulating a company's overall governance performance (Aggarwal et al., 2009; Gupta, Krishnamurti and Tourani-Rad, 2018). This simplification aids stakeholders, including investors and regulatory bodies, in juxtaposing and evaluating governance practices across diverse entities (Ashbaugh-Skaife, Collins and LaFond, 2006; Zhou, 2014). Secondly, amalgamating the four categories into one variable mitigates the risk of oversimplification, fostering a more holistic assessment of CGQ while reducing information asymmetry and enhancing stakeholder trust (Aggarwal et al., 2009). This approach acknowledges the interconnectedness of various governance facets, underscoring the holistic nature requisite for effective governance (Reverte, 2009; Pavlopoulos, Magnis and Iatridis, 2017). Finally, presenting CGQ as a singular variable fosters’ transparency and accountability, furnishing stakeholders with a lucid and readily comprehensible metric of a company's governance practices. This transparency strengthens stakeholder confidence and enhances the firm's reputation, aligning with the broader objectives of good governance (Hail and Leuz, 2006). By consolidating the governance dimensions into a unified measure, our approach provides a practical and robust tool for assessing overall governance performance. This methodology aligns with prior research emphasizing the importance of integrated governance metrics in reducing complexity and improving comparability across firms (Ashbaugh-Skaife, Collins and LaFond, 2006; Aggarwal et al., 2009; Reverte, 2009; Pavlopoulos, Magnis and Iatridis, 2017; Gupta, Krishnamurti and Tourani-Rad, 2018; Al-Gamrh, Farooq and Ahsan, 2024; Honey, Ahsan and Migliori, 2025).”*

We have clarified the definitions of the control variables as requested. Company size (SIZE) is now defined as the natural logarithm of a firm’s market value of equity, and leverage (LEV) is defined as the ratio of total debt to total assets. These updates have been incorporated into Section 2.4. We appreciate your feedback, which has helped improve the clarity of our manuscript.

**Comments #5:** In equation 2, presented on line 460, the interaction term is already included, as well as the control variables, which reduces the usefulness of equation 3 and 4.

Response to comment #5: Thank you for bringing this to our attention. We have revised the equations in accordance with your suggestion. Please see the corrected versions of equations 2 and 3 in the manuscript.

**Comments #6:** Regarding results documented on table 3, authors do not comment the coefficient of interaction term IRQ and CGQ, which is statistically significant at 1% level, but positive, mitigating the reduction of the cost of capital. This result is just mention after, when authors analyze table 4. In any case, an economic argument must be provided for this result. In addition, I suggest to remove from the legend the p values that are not presented in the table (* and **)

Response to comment #6: Thank you for your valuable suggestion. We have revised the section "4.3 Moderating role of CGQ on IRQ disclosure and ICC" in line with your feedback. Kindly refer to the updated text in Section 4.3.

**Comments #7:** Once again, it would be relevant having the rational for weaker CGQ implying lower ICC for each IRQ, as illustrated in figure 4.

Response to comment #7: Thank you for your valuable suggestion. We have revised the interpretation of Figure 4 in the manuscript. Please refer to the highlighted text in the manuscript and the updated explanation provided below.

*“To delve deeper into the intricacies of moderations, we adhered to the guidelines outlined by Cohen et al. (2002) and conducted a simple slope analysis, as recommended by Aiken and West (1991). The slope analysis depicted in Figure 4 illustrates how CGQ moderates the relationship between IRQ disclosure and ICC. The analysis revealed that the relationship between IRQ disclosure and ICC varied significantly depending on the level of CGQ. Specifically, under weak CGQ, there was a steeper negative slope between IRQ disclosure and ICC, indicating that as IRQ disclosure increased, ICC decreased substantially. In contrast, under strong CGQ, the negative association was less pronounced, resulting in a flatter slope. The rationale for weaker CGQ implying a lower ICC, as illustrated in Figure 4, lay in the compensatory role played by high-quality integrated reporting. Firms with weaker governance standards often faced greater investor skepticism due to higher perceived risks, such as agency conflicts or managerial opportunism (Jensen and Meckling, 1976). In such cases, high-quality IRQ disclosures served as a compensatory mechanism, signaling transparency and accountability to investors, thereby reducing ICC more effectively (Dhaliwal et al., 2011; Chen and Zhang, 2021). This compensatory effect diminished when CGQ was strong because robust governance structures already instilled confidence in investors, leading to relatively smaller incremental benefits from improved IRQ disclosure (Zhou, Simnett and Green, 2017; Gupta, Krishnamurti and Tourani-Rad, 2018).*

*Furthermore, Figure 4 indicated that the difference in ICC between low and high CGQ levels was more pronounced when IRQ disclosure was high. This suggested that high IRQ disclosure was particularly impactful for firms with weaker governance, as it directly addressed gaps in investor confidence and mitigated concerns related to information asymmetry. Conversely, when IRQ disclosure was low, the difference in ICC across CGQ levels was smaller, highlighting the limited efficacy of low IRQ disclosure in reducing ICC regardless of governance quality.*

*In conclusion, the regression results and Figure 4 collectively demonstrate that CGQ moderates the relationship between IRQ disclosure and ICC. The compensatory role of IRQ in weaker CGQ settings underscores its critical importance in improving investor perceptions and reducing ICC, particularly in developed markets.”*

**Comments #8:** In line 630, it would be clearer to mention that in table 5, model 1 corresponds to GMM, model 2 to pooled OLS and model 3 to mean quantile regression.

Response to comment #8: Thank you for your valuable comment. We have revised the text in alignment with your suggestion.

**Response to Reviewer #2 comments**

**Comments #1:** First, please provide more detailed descriptions of data sources and processing procedures: Ensure the reliability of the data and the transparency of the methodology.

Response to comment #1: Thank you for your valuable comment. The data for this study were sourced from a combination of publicly available databases, company annual reports, regulatory filings, and institutional shareholder services (ISS) risk metrics. Specifically, integrated reporting quality data were derived from content analyses of firms’ annual reports and sustainability reports, following standardized disclosure frameworks such as the IIRC guidelines. CGQ data were obtained from ISS, which aggregates governance metrics based on filings, corporate websites, and independent assessments. To ensure data reliability, we cross-validated the collected data with secondary sources, including company websites and third-party governance assessment platforms.

Data processing involved standardizing the variables across firms to enable comparability. For example, IRQ scores were calculated using a weighted scoring method that evaluated disclosures on financial, environmental, social, and governance dimensions. CGQ scores were derived by aggregating the four ISS governance categories, as previously described. Additionally, financial data, including ICC, leverage, firm’s market value of equity, long-term growth, return on assets, CSR, and ratio of total debt to total assets, were retrieved from Thomson Reuters Eikon and I/B/E/S within the DataStream databases, ensuring the accuracy and timeliness of the variables. The dataset spans the period from 2018 to 2022, covering top-listed firms in Australia and New Zealand.

After collecting the data, we performed a series of preprocessing steps, including outlier detection and removal to ensure robustness. Missing data points were addressed using multiple imputation techniques where feasible, consistent with prior research practices (Rubin, 2004). To further validate the dataset, we conducted descriptive analyses, ensuring that key variables met the assumptions required for statistical analyses.

We have described the data sources and processing procedures in detail in the revised manuscript. Please refer to Sections 2.4, 3.1, 3.2.1, 3.2.2, and 3.2.3 for further clarification.

**Comments #2:** Second, please explain why the firm-level fixed effect is not included in the models, and enhance the detail and explanation in the results section.

Response to comment #2: We sincerely apologize for the oversight and acknowledge that this information was inadvertently omitted in the earlier version of the manuscript. In our baseline model, we used company and year fixed effects, and this has now been appropriately incorporated into the revised manuscript. Please refer to the updated Equations 2 and 3, as well as Table 3, which reflect these changes.

In response to the comment on the detailed explanation of the results section, we have revised it accordingly. Please refer to the updated Section 4 for these revisions.

Thank you once again for your precious comments and advice. Those comments are all valuable and very helpful for revising and improving our manuscript. We have revised the manuscript accordingly, and our point-by-point responses are presented above. We hope you are satisfied with our answers and the new data we have provided. Our deepest gratitude goes to you for your careful work and thoughtful suggestions that have helped improve this paper substantially.

Sincerely yours

*All Authors’*
